# When It’s Not Cancer: An Uncommon Case of Tubular Ectasia of the Epididymis as a Post-vasectomy Sequela

**DOI:** 10.7759/cureus.94316

**Published:** 2025-10-10

**Authors:** Hassan Mahbuba, Zamanali Khakhar, Safiyah Shah, Nidhi Leekha, Sayed K Ali

**Affiliations:** 1 College of Medicine, University of Kufa, Kufa, IRQ; 2 School of Medicine, University of Nairobi, Nairobi, KEN; 3 Radiology, Aga Khan University Hospital, Nairobi, KEN; 4 Medicine, Aga Khan University Hospital, Nairobi, KEN

**Keywords:** epididymis, post-vasectomy complications, testicular mass, tubular ectasia, vasectomy

## Abstract

Tubular ectasia of the epididymis is a rare, benign condition that arises from blockage of the seminiferous ducts, resulting in dilated tubular structures and retention of sperm within the epididymis. It represents an important differential among benign intratesticular lesions and should be considered in the diagnostic evaluation of scrotal pathologies. Recognition of its typical imaging characteristics, in conjunction with relevant clinical history, enables accurate identification and prevents unnecessary diagnostic or therapeutic procedures. This case report describes a 43-year-old male patient with post-vasectomy tubular ectasia, highlighting the diagnostic approaches utilized and management undertaken.

## Introduction

Tubular ectasia of the epididymis is an atypical and seldom-reported outcome that may arise secondary to various underlying etiologies, most notably in individuals with a history of vasectomy, but it can also occur in association with other causes of obstruction of the ductus deferens [1]. It represents a benign intratesticular lesion and involves the gradual and sustained accumulation of sperm due to obstruction of the vas deferens, resulting in elevated tubular pressure, dilation, and cystic changes within the epididymis [2]. Its clinical significance stems from its potential to mimic malignant testicular tumors on sonographic imaging, which can result in considerable psychological distress for patients and may prompt unnecessary invasive interventions [3]. This highlights it as an important differential among clinicians and radiologists.

Herein, we present a case of a 43-year-old male patient with post-vasectomy tubular ectasia. As benign intratesticular tumors account for only about 5% of all intratesticular tumors and are therefore uncommon, accurately identifying these lesions is crucial to distinguish them from potentially malignant testicular neoplasms [2]. Tubular ectasia of the epididymis is a recognized finding following vasectomy, and this case aims to contribute to the limited existing literature by illustrating its clinical presentation, diagnosis, and management approach.

## Case presentation

A 43-year-old male patient with no significant past medical history presented to the clinic with a three-month history of progressively worsening scrotal pain, which was exacerbated by physical exertion. The patient was an avid runner, typically covering approximately 10 kilometers every other day, and cycled on alternate days. He denied any history of smoking or tobacco use and had no family history of cancer. Notably, he had undergone a vasectomy two years prior as a form of contraception.

On examination, the patient appeared comfortable and in no acute respiratory distress, with vital signs within normal limits. Cardiopulmonary and abdominal examinations were unremarkable. Genitourinary examination revealed a circumcised male with bilaterally descended testes that felt slightly irregular with a bumpy texture but were non-tender, and no inguinal hernias were detected.

**Figure 1:**
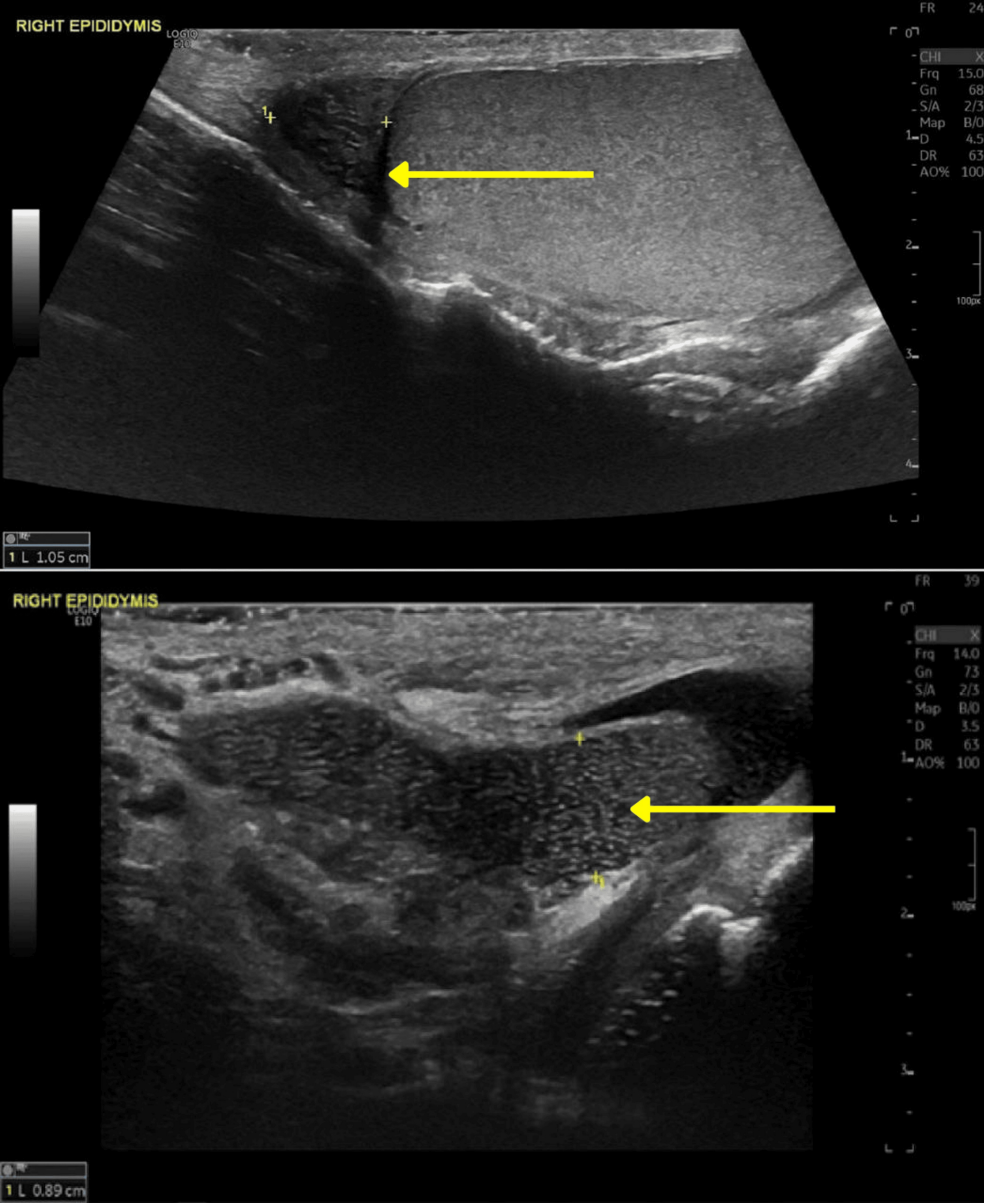
Images of the enlarged right epididymis in longitudinal view demonstrating 'speckled' appearance due to multiple interfaces between the epididymal walls and fluid (yellow arrow).

**Figure 2:**
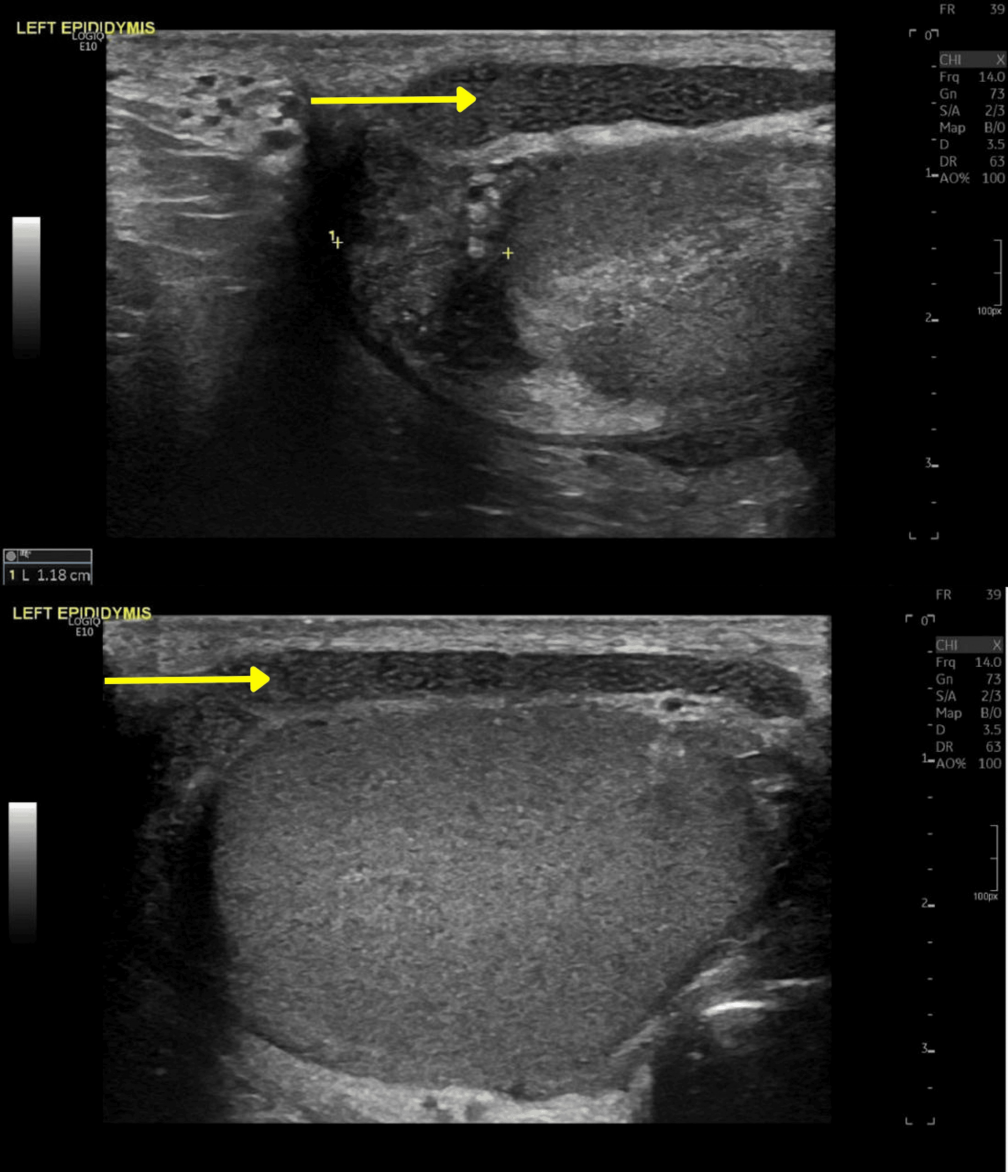
Images of the enlarged left epididymis in longitudinal view demonstrating 'speckled' appearance due to multiple interfaces between the epididymal walls and fluid (yellow arrow).

Laboratory studies demonstrated normal renal function (creatinine). Tumor markers, including alpha-fetoprotein (AFP), lactate dehydrogenase (LDH), and beta-human chorionic gonadotropin (β-HCG), were all within normal limits. A CT scan of the abdomen was unremarkable. However, scrotal ultrasonography revealed bilateral grade II varicoceles and findings consistent with bilateral epididymal tubular ectasia, with a "speckled" appearance of the epididymis (Figures 1, 2).

The patient was provided with reassurance regarding the benign nature of the findings. He was counseled to moderate his vigorous cycling and high-intensity workouts to reduce mechanical strain on the scrotal contents. The use of supportive undergarments and analgesics was advised to relieve discomfort and provide additional scrotal support. These measures collectively contributed to the alleviation of pain and improvement of symptoms. Additionally, he was advised to seek prompt medical attention if he developed any new or worsening symptoms, including localized pain, increased pressure, or swelling.

## Discussion

The epididymis is a curved, comma-shaped structure attached to the posterior surface of the testes. It consists of compact and highly convoluted tubes and consists of three parts: the head, body and tail. The head is connected to the upper pole of the testes and receives seminal fluid from the ducts of the testes. It permits storage, maturation, and transport of spermatozoa into the distal portion of the epididymis [4].

Tubular ectasia of the epididymis is a rare, benign condition characterized by cystic dilatation of the epididymal tubules [2]. The development of tubular ectasia is thought to result from altered sperm transport and increased intraluminal pressure within the epididymal tubules after vasectomy, leading to their gradual distention and cystic transformation [1,5]. Because of this strong association, tubular ectasia is often regarded as a characteristic sonographic finding in post-vasectomy patients. Less commonly, tubular ectasia may arise secondary to other causes of vas deferens obstruction, including infections, trauma, or previous surgical interventions [6].

Tubular ectasia is predominantly observed in patients over the age of 55 [3], although it can be detected earlier in those with relevant clinical history. It is frequently discovered incidentally on scrotal ultrasound but may also present with nonspecific symptoms, such as dull, aching scrotal pain, a dragging sensation, or a soft, palpable scrotal mass.

The initial investigation of choice for diagnosing ectasia of the epididymis is ultrasonography. Classical ultrasound findings include linear hypoechoic structures with echogenic walls within enlarged epididymides, representing dilated efferent tubules and producing a characteristic "speckled" appearance of the epididymis [5]. An additional finding on ultrasound includes “dancing megasperm,” which appears as continuously oscillating, minuscule, echogenic foci within dilated tubules of the epididymis. This sign is commonly seen in post-vasectomy patients and others with obstruction of the spermatic cord, and it is thought to represent clusters of trapped spermatozoa that oscillate due to turbulence generated during scrotal ultrasound [7]. On colour doppler sonography, tubular ectasia appears hypovascular [8].

Due to its non-specific clinical presentation, the differential diagnosis for epididymal tubular ectasia is broad. Several benign scrotal lesions must be considered and systematically ruled out through thorough clinical assessment and scrotal ultrasonography. These include hydroceles, spermatoceles, epididymal cysts, and most notably, varicoceles [2]. Varicoceles are a key differential diagnosis for tubular ectasia of the epididymis. The two can be distinguished on color Doppler ultrasonography, as tubular ectasia demonstrates no vascular flow, whereas a varicocele shows venous flow [8].

More importantly, it is critical to rule out malignant scrotal lesions during the evaluation of testicular abnormalities [3]. Testicular cancer is the most common malignancy in males aged 15-45 years, and its incidence has been steadily rising [9]. Although tubular ectasia and testicular cancer can present clinically with overlapping symptoms, such as scrotal discomfort or palpable masses, careful clinical examination combined with scrotal ultrasonography can differentiate the two. Tubular ectasia demonstrates characteristic imaging findings that are distinct and can be easily differentiated from testicular malignancies, which present as solid, hypoechoic intratesticular masses [1,10].

There are currently no standardized guidelines for the management of tubular ectasia of the epididymis, largely due to its rarity and typically benign, asymptomatic nature. As a result, management is generally conservative with reassurance and symptomatic relief with analgesics. However, in patients with persistent pain or functional impairment despite conservative measures, surgical options such as epididymectomy have been described as an effective treatment strategy [11].

## Conclusions

Tubular ectasia of the epididymis, though uncommon, is a recognized complication of vasectomy and may present with vague or nonspecific symptoms such as scrotal discomfort or pain. Its radiologic appearance can closely mimic that of malignant intratesticular lesions, often causing significant anxiety and sometimes leading to unnecessary interventions. A major challenge in managing this condition is its limited representation in the literature and the absence of standardized treatment guidelines. Accurate diagnosis depends on careful correlation of clinical history, physical examination, and high-resolution ultrasonography, which remain the most effective tools for distinguishing tubular ectasia from malignant pathology. Conservative management in this patient led to sustained symptom relief and clinical stability, highlighting its effectiveness as a viable and successful therapeutic approach in management of this condition. Greater awareness and recognition of this benign entity are essential to help clinicians avoid misdiagnoses, reduce overtreatment, and provide patients with appropriate reassurance and management.
